# The Chemical Likelihood of Ribonucleotide-α-Amino acid Copolymers as Players for Early Stages of Evolution

**DOI:** 10.1007/s00239-019-9887-7

**Published:** 2019-02-20

**Authors:** Ziwei Liu, Ghinwa Ajram, Jean-Christophe Rossi, Robert Pascal

**Affiliations:** 1 IBMM, University of Montpellier, CNRS, ENSCM, Montpellier, France; 2 Present Address: MRC Laboratory of Molecular Biology, Cambridge Biomedical Campus, Cambridge, CB2 0QH UK

**Keywords:** RNA, α-Amino acid, Copolymer, Translation, Aminoacylation, Phosphoramidate

## Abstract

How ribosomal translation could have evolved remains an open question in most available scenarios for the early developments of life. Rather than considering RNA and peptides as two independent systems, this work is aimed at assessing the possibility of formation and stability of co-polymers or co-oligomers of α-amino acids and nucleotides from which translation might have evolved. Here we show that the linkages required to build such mixed structures have lifetimes of several weeks to months at neutral pH and 20 °C owing to the *mutual protecting effect* of both neighboring phosphoramidate and ester functional groups increasing their stability by factors of about 1 and 3 orders of magnitude, respectively. This protecting effect is reversible upon hydrolysis allowing the possibility of subsequent reactions. These copolymer models, for which an abiotic synthesis pathway is supported by experiments, form a basis from which both polymerization and translation could have logically evolved. Low temperatures were identified as a critical parameter for the kinetic stability of the aminoacylated nucleotide facilitating the synthesis of the model. This observation independently supports the views that any process involving RNA aminoacyl esters, outstandingly including the emergence of translation, was more probable at 0 °C or below and might be considered a kinetic marker constraining the environment in which translation has evolved.

**Electronic supplementary material:**

The online version of this article (10.1007/s00239-019-9887-7) contains supplementary material, which is available to authorized users.

## Introduction

The idea that the origin of life could result from a replication of the sequence of a biopolymer was a logical consequence of the discovery of the double helix structure of DNA (Watson and Crick 1953). This discovery emphasized the role of nucleic acids in the storage of information and as a main driver of life and its developments. By contrast, the results of Miller’s experiment (Miller 1953), published three weeks later, pointed toward the formation of α-amino acids under prebiotic conditions and therefore toward an easier synthesis of peptides, the other main class of biopolymers. However, the chemical replication and inheritability of peptide sequences without translation is unsolved yet. On the other hand, the possibilities of template replication of nucleotides as well as the role of RNA in biochemistry pointed toward a major contribution of RNA in early life (Rich 1962; Crick 1968; Orgel 1968; White 1976). Unfortunately, the abiotic synthesis of long strands of RNA capable of playing the role of polymerase has not been successful to date even though chemical pathways for the abiotic synthesis of nucleotides have been discovered (Anastasi et al. 2006; Powner et al. 2009; Becker et al. 2016). Following the hypothesis of an RNA world, the transition of the RNA world to an RNA–protein world, a subsequent stage of evolution, requires a common chemistry of RNA and amino acids or peptides (Szathmáry 1999). Since α-amino acids were likely present on the primitive Earth, it would be surprising if the corresponding chemistry could not have played a role at earlier stages eventually leading to both RNA and coded peptides.

**Scheme 1:**
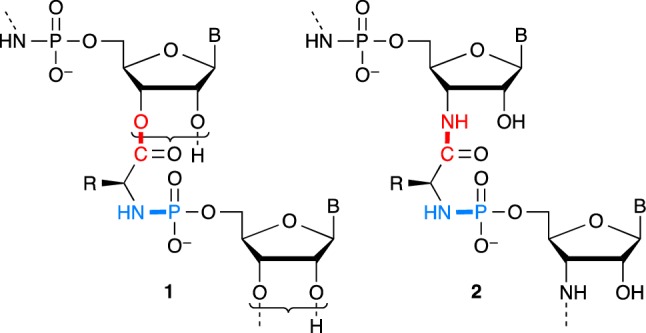
Structure of co-oligomers of α-amino acids and ribonucleotides **1** involving carboxylic acid esters and phosphoramidates as key linkages and the stable amide structural analogues of 3′-amino-3′-deoxy-nucleotides **2** studied by Zielinski and Orgel (1989). The presence of a mixture of 2′- and 3′-regioisomers at equilibrium for structure **1** is symbolized in the scheme by a curly bracket

One of the chemical possibilities offered by the common chemistry of α-amino acids and nucleotides involves the formation of phosphoramidate bonds, which has been suggested as relevant to prebiotic chemistry in several instances (Jauker et al. 2015; Griesser et al. 2017a, b; Ni et al. 2009). In an attempt to enlarge this approach, we decided to investigate the potential role of copolymers of α-amino acids and ribonucleotides **1** containing phosphoramidate and ester linkages (Scheme 1) as an alternative to the limitations of both peptide and RNA worlds. One of the advantages of these structures is the foreseen effectiveness of formation of a phosphoramidate linkage compared to that of phosphodiesters demonstrated by the group of Orgel (Lohrmann and Orgel 1976a; Zielinski and Orgel 1985). The reaction of nucleotides having a 3′-hydroxyl group substituted for an amino group easily produces phosphoramidate linkages upon activation by 1-ethyl-3-(3-dimethylaminopropyl)-carbodiimide (EDC) and served as a tool to investigate template replication (Zielinski and Orgel 1985, 1987a, 1987b; von Kiedrowski et al. 1991; Sievers and von Kiedrowski 1994; Kaiser et al. 2012; Zhang et al. 2013). Interestingly, the corresponding—amide-linked—aminoacylated nucleotide modified by an amino group **2** (Scheme 1) also yielded efficiently phosphoramidates upon reaction with nucleotides (Zielinski and Orgel 1989). Orgel et coll. (Shim et al. 1974) even observed the formation of sequences corresponding to **1** from an—ester linked—2′(3′)-glycyl-5′-AMP. It is worth noting that the presence of a template was reported to greatly facilitate the reaction and the authors concluded in wondering about the potential role of oligomers of that kind in the context of prebiotic chemistry.

The ester bond in the copolymer is analogous to that of aminoacyl-tRNAs (aa-tRNAs) known as the unstable activated species of peptide biosynthesis and that are present as an equilibrium mixture of 2′- and 3′-isomers (Wolfenden et al. 1964). Taking into account the fact that removing the positive charge of the aminoacyl moiety increases the kinetic stability of the ester bond in aa-tRNAs by two orders of magnitude (Wolfenden 1963; Schuber and Pinck 1974) and that this effect could be enhanced by the negative charge of the phosphoryl substituent, we deduced that the corresponding phosphoramidates could present a time stability suitable for allowing them to play a significant role in chemical evolution. To the best of our knowledge, the pioneering work of Shim et al. (1974), mainly motivated by the identification of structures capable of template replication, an ability that was not confirmed later using closely related amide-based derivatives **2** (Zielinski and Orgel 1989), has not been investigated further. On the basis of our earlier experience in aminoacylation using α-amino acids strongly activated under the form of 5(4*H*)-oxazolones and *N*-carboxyanhydrides (NCAs) (Liu et al. 2014, 2016a, 2016b), we embarked on a study of potential prebiotic pathways of formation of phosphoramidate-linked adducts as well as of their hydrolytic stability. We now report the first results demonstrating that abiotic pathways for the formation of both ester and phosphoramidate key linkages of structure **1** are indeed available and that their kinetic stability is compatible with a role of chemical intermediates in a system based on amino acids and nucleotides predating the better established RNA–protein world. Hypothetical pathways through which chemical evolution could have ensued are additionally proposed to illustrate the possible contribution of these structures to the early stages of life from which translation and replication could have evolved at the same time without requiring a stage based on RNA only.

## Results and Discussion

**Scheme 2:**
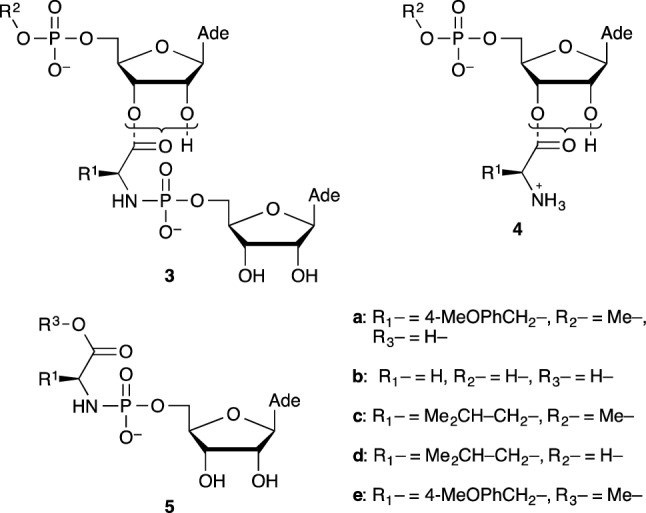
Structures of α-amino acid—ribonucleotide adducts mentioned in this work

The study of the co-oligomers was undertaken using the simple trimer models **3a** (with a Tyr(Me) amino acid residue selected for analytical purpose (with a significant absorbance at 273 nm that becomes almost negligible at the 248 nm minimum used for the analysis of adenine-containing species) and likely to be representative of most residues without a reactive side-chain) containing the key phosphoramidate and ester linkages and a 5′-terminus blocked by a methyl group (Scheme 2). AMP and its methylated derivative were used as models of reactivity for mononucleotides and RNA 3′-end, respectively. Coupling reactions were carried out using EDC, well known to be efficient for the phosphoramidate ligation of nucleotides (Zielinski and Orgel 1985, 1987a, 1987b, 1989; von Kiedrowski et al. 1991; Sievers and von Kiedrowski 1994; Kaiser et al. 2012; Zhang et al. 2013). Though it does not represent a plausible reagent in a prebiotic environment, it was considered here as a practical laboratory model of more relevant and maybe more efficient activation systems required for the abiotic formation of any kind of biopolymers (including notably RNA) in the origin of life process.

Using ^14^C-labeled substrates, Shim et al. (1974) were able to detect the formation of a similar phosphoramidate adduct **3b** in up to 80% yield from a reaction of 12.5 mM 2′(3′)-Gly-5′-AMP ester **4b** with the 12.5 mM 5′-AMP-imidazolide [used for decades as an activated nucleotide in experiments on spontaneous or template directed polymerization as well as other more efficient 5-substituted derivatives of imidazole (Li et al. 2017)] in the presence of 200 mM NaCl and 75 mM MgCl_2_ and 50 mM poly-U as a template for oligomerization at 0 °C. The authors also investigated the reaction of the glycine phosphoramidate of 5′-AMP **5b** with 5′-AMP in the presence of EDC as an activating agent, which also indicated the formation of a similar product in up to 43.5% in the presence of poly-U and imidazole.

### Formation of the Phosphoramidate Linkage

**Fig. 1:**
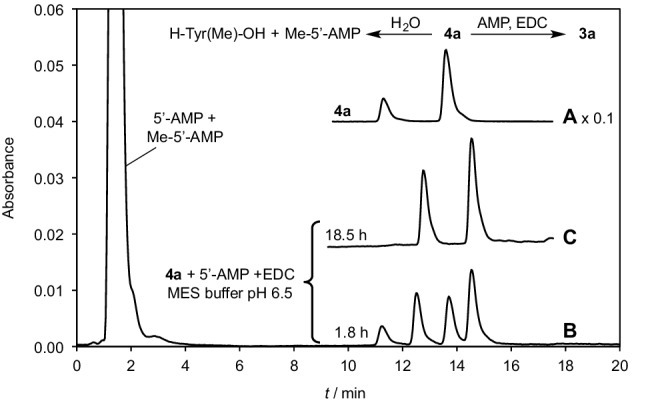
Reaction of 5 mM ester **4a**, 5 mM 5′-AMP and 500 mM EDC in a 100 mM MES buffer pH 6.5; HPLC chromatograms of samples of the reaction medium (20 µL diluted to 1 mL with water, method A, detection 248 nm at the minimum of the uv spectrum of Tyr(Me)); *A* solution of unreacted ester **4a** in water; *B* reaction medium after 108 min of reaction; *C* after 18.5 h

We investigated phosphoramidate formation from the free 5′-AMP nucleotide that was activated and reacted with the aminoacylated methylated 5′-AMP model **4a** (Fig. 1) in the presence of the EDC reagent. This procedure proved to be convenient and yielded an adduct in moderate yield indicating that phosphoramidate formation is able to compete with the hydrolysis of the ester at reasonably low concentrations (Fig. 1). For instance, a solution containing 5 mM 5′-AMP, 5 mM ester **4a** and 500 mM EDC in a 100-mM MES buffer (pH 6.5) was allowed to react at room temperature and monitored by HPLC (Fig. 1). The selected HPLC method (Method A, Supporting Information) showed the presence of the two regioisomers (retention times 11.2 and 13.6 min in a 3:7 ratio) of the starting material **4a** and the addition of EDC induced the formation of two new HPLC peaks (retention time 12.6 and 14.5 min in a 4:6 ratio). After 18.5 h, the starting ester was totally consumed and the mixture was analyzed by HPLC-MS indicating the presence of two isomers with a mass corresponding to that of co-trimer **3a** (HPLC-MS, retention time 1.55 and 1.58 min, Method D, Supporting Information, negative mode, *m*/*z* calculated for C_31_H_38_N_11_O_15_P_2_^−^ : 866.2030, found 866.20). The reaction was continued for 300 h without major change, and the final yield was estimated to approximately 16% making the hypothesis that the UV absorption of the adenine moiety in the chromatogram is not modified by the reaction, which is reasonable with respect to the accuracy of the assessment. At that time, the medium was acidified to pH 2 in order to confirm the presence of a phosphoramidate linkage in the regioisomers of co-trimer **3a** the and the HPLC analysis was repeated after 3 days indicating that the hydrolysis of the adduct reverted the starting ester **4a** as two regioisomers (HPLC-MS, retention time 1.54 and 1.56 min, Method D, Supporting Information, negative mode, *m*/*z* calculated for C_21_H_26_N_6_O_9_P^−^: 537.1504, found 537.10). The product’s reversion in acid to the starting material is consistent with the presence of an acid-sensitive phosphoramidate linkage and with the attribution of the structure of co-trimer **3a** to the adduct, which is in agreement with the presence of two regioisomers as well. A similar reaction was carried out in D_2_O and analyzed by ^31^P-NMR (Supporting Information Figure S1) indicating the presence of two signals at 5.99 and 6.27 ppm supporting the presence of isomers bearing a phosphoramidate moiety.

The yield of the reaction could be substantially increased by performing the reaction at 4 °C over longer periods with yields approaching the one published by Shim et al. (1974). A rationale for this observation is that the hydrolysis of ester **4a** occurring at the same time as phosphoramidate formation is responsible for the low yield of co-trimer **3a** at 25 °C and can be overcome owing to the unusually high influence of temperature on the rates of hydrolysis of ester **4a** by reducing the temperature to a value close to 0 °C. Owing to its time stability increased compared to aminoacylated nucleotide **4a**, the adduct **3a** could be separated by preparative chromatography in triethylammonium acetate buffers with an overall yield exceeding 50% and characterized by NMR and MS (Supporting Information). The targeted basic linkage present in polymer **1** could therefore be obtained through an abiotically plausible process provided that aminoacylated derivatives of ribonucleotides analogous to **4a** are available.

### Aminoacylation

**Scheme 3:**
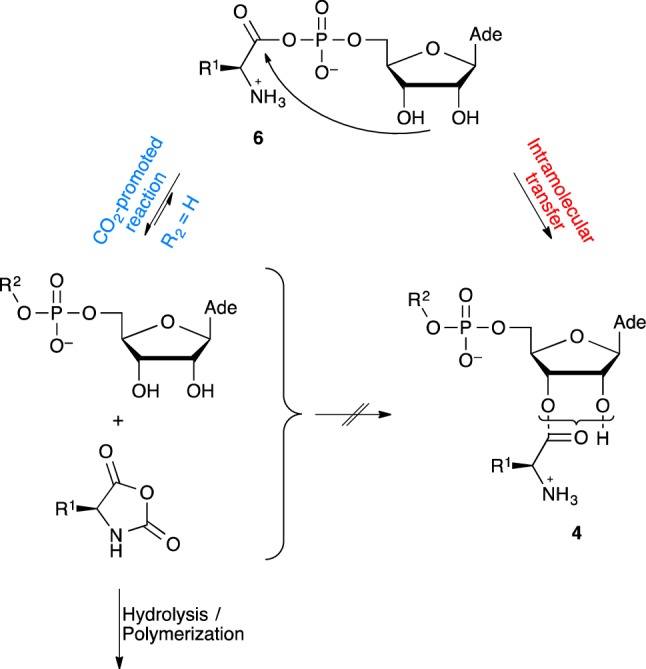
Inefficiency of the direct aminoacylation of nucleotides 5′-AMP (*R*^2^ = H) and Me-5′-AMP (*R*^2^ = Me) by α-amino acid *N*-carboxyanhydrides (Liu et al. 2016b) can be overcome for the former through the intramolecular aminoacyl transfer from adenylates (Wickramasinghe et al. 1991) like **6d** (R^1^ = Me_2_CHCH_2_), which can compete with the breakdown of the mixed anhydride promoted by CO_2_ (the reverse of the aminoacylation pathway based on the reaction of NCAs that is fast in the presence of CO_2_)

Although procedures for chemically aminoacylating ribonucleotides have been proposed, we considered the possibility of a reaction involving α-amino acid *N*-carboxyanhydrides (NCAs) as activated reagents and an intramolecular aminoacyl transfer (Scheme 3) for reasons that are listed below. Firstly, and from a synthetic perspective, imidazolides of *N*-protected amino acids turned out to be efficient for aminoacylation (Profy and Usher 1984a, b) though their occurrence in an abiotic context is questionable and the presence of a protecting group removes to the process most of its relevance to our present goal. Ribozymes active in aminoacylation and using adenylates, active esters or thioesters have been selected (Illangasekare et al. 1995, 1997; Lee et al. 2000). In an origin of life context, it is worthy to note that very short sequences proved to be active (Turk et al. 2010, 2011). However, strongly activated α-amino acid derivatives such thioesters (Brack 1987) as well as aminoacyl adenylates (Wickramasinghe et al. 1991; Liu et al. 2014) having a free amino group are not stable in aqueous environments in the presence of CO_2_ and the lifetime of adenylates at the present day concentration of bicarbonate in the ocean or in biological media has been assessed to a few seconds only (Liu et al. 2014), removing any utility of amino acid phosphate anhydride as activated precursors for the formation of peptides in a prebiotic environment richer in CO_2_. The CO_2_-promoted reaction, which strongly increases peptide formation (Liu et al. 2014), takes place through an NCA intermediate (Scheme 3), which can then be hydrolyzed or polymerized. No advantage can therefore be found for adenylates as peptide precursors, which raises the question of their selection as essential intermediates of the biosynthesis of peptides. The important role of adenylates and other mixed anhydrides could, however, lie in the fact that NCAs proved to be inefficient for the aminoacylation of methylated 5′-AMP (Me-5′-AMP), a simple model of the reactivity of RNA 3′-end (Liu et al. 2016b). This behavior, contrasting with that of other reagents like 5(4*H*)-oxazolones as activated peptide segments, was considered as resulting from a different reaction path avoiding any assistance from the vicinal diol of the ribose moiety (Liu et al. 2016b).

We therefore considered the Lacey’s group invaluable observation (Wickramasinghe et al. 1991) of the occurrence of an intramolecular aminoacyl transfer in aminoacyl adenylates allowing the formation of esters at the 2′(3′)-ribose moiety **4** from the mixed anhydride **6** (Scheme 3). The reaction was reported to be highly effective at pH values below 6 provided that CO_2_ is rigorously excluded from the reaction medium (Wickramasinghe et al. 1991). Moreover, the authors observed a high degree of stereoselectivity in favor of the natural configuration—a d-ribose moiety induced a preference for aminoacylation by l-amino acids—supporting an important role for this process in the emergence of the chiral coupling between the two classes of biomolecules (Wickramasinghe et al. 1991). Though this purely chemical process presents the drawback of being largely surpassed by the fast CO_2_-promoted hydrolysis of adenylates, even at low contents of this gas in the atmosphere (Wickramasinghe et al. 1991), we realized that the reverse path, the formation of adenylates from NCAs, would also take place (Liu et al. 2014; Biron et al. 2005; Leman et al. 2006) provided that other synthetic processes could deliver α-amino acid *N*-carboxyanhydrides in the medium. Therefore, the possibility of aminoacylation of nucleotides could involve an indirect rather than direct reaction of NCAs, reagents for which prebiotically plausible pathways of formation have been identified (Pascal et al. 2005; Danger et al. 2006; Leman et al. 2004).

**Fig. 2:**
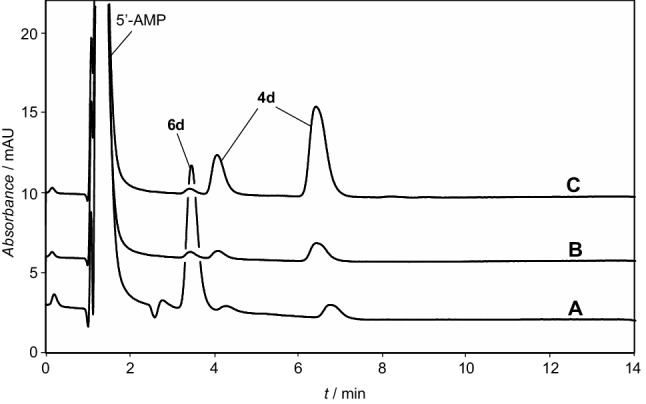
Reaction of Leu-NCA (25 mM overall added in 5 portions over 4 h) with 1 mM 5′-AMP in a 100 mM MES buffer (pH 5.5) containing 50 mM MgCl_2_ at room temperature. HPLC monitoring of the reaction progress (Method C, detection 248 nm): *A* reaction medium after the addition of the last portion of Leu-NCA at 4 h; *B* reaction medium at 24 h (1.9% total yield of esters **4d**); *C* reaction medium at 24 h after addition of ester **4d**. Identification of HPLC peaks: 1.3 min, 5′-AMP, 3.5 min leucyl adenylate **6d**, and 4.1 and 6.4 min, 2′- and 3′-regioisomers of ester **4d**, respectively

Taking into account our previous observation that the decomposition of aminoacyl adenylates into NCAs promoted by CO_2_ is reversible (Liu et al. 2014), we therefore investigated the possibility that the NCA could react with 5′-AMP to yield a mixed anhydride at equilibrium, which could be subsequently trapped by intramolecular transfer (Scheme 3). This procedure worked properly, albeit in low yield (Fig. 2). Two HPLC peaks (retention time 4.1 and 6.4 min, Method C) corresponding to the 2′(3′)-regioisomers of the aminoacylated species **4d** were indeed observed by reaction of Leu-NCA (5 additions of a total of 25 mM over 4 h to reduce the occurrence of NCA polymerization) with 5′-AMP at pH 5.5 (100 mM MES buffer). A ca. Twofold increase in yield could be observed in the presence of 50 mM Mg^2+^, and the intramolecular nature of the reaction could be confirmed by the absence of reaction of the methylated ester **4c** (Supporting Information) (Liu et al. 2016b).

The intramolecular nature of the aminoacylation process, already supported by experiments which was carried out at very low concentration (Wickramasinghe et al. 1991), is confirmed here by the reaction of the NCA with 5′-AMP, which contrasts sharply with the absence of reaction observed earlier for Me-5′-AMP in which the phosphodiester moiety is unable to give a di-anion and therefore to yield a mixed anhydride (Supporting Information) (Liu et al. 2016b). We previously noticed that the fast decomposition of amino acid-nucleotide mixed anhydrides observed even at low concentration of CO_2_ removes any utility as peptide precursors to these reagents since the NCA intermediates behave as active monomers in a much better way (Liu et al. 2014). Their early role in chemical evolution could therefore have been to act as aminoacylating agents rather than as activated precursors of peptides. The possibility of an intramolecular transfer of the aminoacyl moiety from adenylate anhydrides to the 3′-hydroxyl group is an additional example of the importance of intramolecular reactions in a prebiotic context and of *induced intramolecularity* for the emergence of catalysis (Pascal 2003, 2015).

### Kinetical Study of the Stability of the Phosphoramidate Linkage and of 2′(3′)-Aminoacylated Nucleotides

**Fig. 3:**
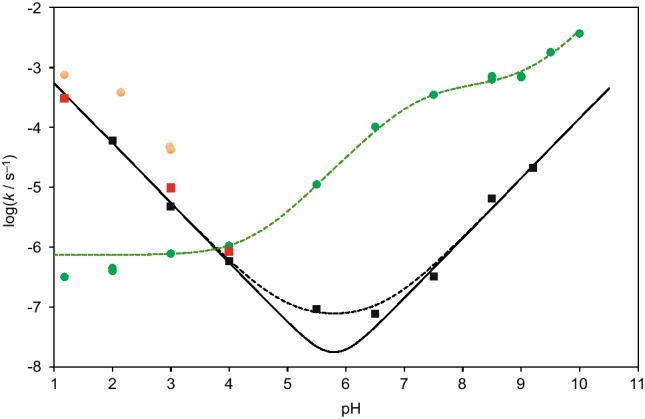
pH-rate profiles for the hydrolytic reactions of the copolymer model **3a** (black squares). Data for the ester hydrolysis of 2′(3′)-leucyl-5′-methyl-AMP **4c** (green circles) as well as for the breakdown of the acid-sensitive phosphoramidate linkage of the adduct of methylated tyrosine **5a** (orange circles) and its methyl ester **5e** (red squares) are also provided. The curves for the hydrolysis of co-trimer **3a** were fitted to specific acid and base catalyzed pathways (black line) and adding a pH-independent term (black dotted line). The curve for aminoacyl ester **4c** was fitted considering the hydroxide ion reaction of both the uncharged and protonated forms at the amino group and a pH-independent process for the protonated form in the acid range (green dotted line). The rate constant and p*K*_a_ values used in the calculation are provided in Supporting Information. (Color figure online)

The kinetic stability of copolymer structure **1** is an essential parameter of its possible contribution in early evolution. In aqueous solution, at the pH values used in the experiments, the hydrolysis of the selected model **3a** was monitored by HPLC (retention time of 2′- and 3′-regioisomers 12.4 min and 14.4 min, Method A) in aqueous buffers at different pH values (Fig. 3). By contrast with the cleavage of the acid sensitive phosphoramidate moiety at pH 2 described in the above section on phosphoramidate formation, ester hydrolysis was observed at high pH values as for instance in a 50 mM borax buffer (pH 9.2) where two products were identified by HPLC-MS (method D) after 9 days of reaction as Me-5′-AMP (retention time 1.27 min, Supporting Information, negative mode, *m*/*z* calculated for C_11_H_15_N_5_O_7_P^−^: 360.0715, found 360.07) and the phosphoramidate **5a** (retention time 1.73 min, Supporting Information, negative mode, *m*/*z* calculated for C_20_H_24_N_6_O_9_P^−^: 523.1348, found 523.13). In a way consistent with the presence of two hydrolysis sites, the pH-rate profile of Fig. 3 is mainly characterized by two linear domains corresponding to the acid-catalyzed phosphoramidate hydrolysis at pH values below 5 and to the base-catalyzed cleavage of the ester above pH 6.5. A deviation from calculated curves may indicate the occurrence of a pH-independent ester hydrolysis at intermediate pH values. This pH-rate profile was compared with that of analogues having a simpler structure, namely the aminoacylated RNA model **4c**, the phosphoramidate **5a** and the corresponding methyl ester **5e** (Fig. 3), which were also subjected to hydrolysis under similar conditions. The pH-rate profile for the aminoacylated nucleotide **4c** was similar to the behavior published in the literature for aminoacylated-tRNAs (Wolfenden 1963; Schuber and Pinck 1974) that is characterized by a slower hydrolysis of the neutral aminoacyl moiety predominant at high pH compared to the protonated one (by a factor of ca. 2 orders of magnitude). A similar but stronger influence of the negative charge of the phosphoramidate group was observed for the co-trimer **4c** since the ester proved to be stabilized by a factor exceeding 3 orders of magnitude compared to esters with a protonated free amino group at pH 6.5. This observation means that the ester bond of a 2′(3′)-aminoacylated nucleotide, considered as an aminoacylating agent for protein biosynthesis, is stabilized upon phosphorylation, having lifetimes reaching several weeks or months at moderate temperatures and within a pH range 5–7 anticipated for most early Earth aqueous environments, so that it could constitute the potential building block of a reactive biopolymer having a lifetime sufficient to play a role in the early developments of life. It is worth mentioning that the lifetime of these species could be increased to values measured in years at temperature close to the melting of ice, which were above noticed to increase substantially the yield of phosphoramidate formation.

In the acid range (1 ≤ pH ≤ 3), the breakdown of the copolymer model was above observed to take place at the acid-sensitive phosphoramidate linkage. A comparison was made with the phosphoramidate model **5e** involving a methyl ester instead of a second AMP moiety. No significant difference was observed in the kinetics indicating that the adenosine moiety has a limited influence on phosphoramidate hydrolysis. However, a higher sensitivity toward hydrolysis was observed for the compound **5a** having a free carboxyl group and that is produced by hydrolysis of the whole copolymer model **3a**. This faster hydrolysis of the acid by a factor of c.a. one order of magnitude can be accounted for by an intramolecular pathway involving the free carboxy group that is already well documented for this kind of derivatives (Benkovic and Benkovic 1967; Sampson et al. 1973; Ora et al. 2007).

Finally a special mention must be made on the influence of temperature on the rates of hydrolysis of esters **4a** and **4c**, which is responsible for the increase in yield observed above for the formation of phosphoramidate **3a** at low temperature. The half-life of ester **4c** hydrolysis measured at pH 6.5 turned out to be increased from 3 to 85 h by lowering the temperature from 25 to 0 °C. This influence of temperature on the hydrolysis rates (a 29-fold increase much higher than the 5.7-fold deduced from the empiric rule that rates increase by a factor of twofold every 10 °C (Wolfenden et al. 1999)) is sufficient to explain why low temperatures favor the formation of phosphoramidate **3a**. ^1^H and ^13^C NMR experiments carried out at 2 °C using **4c** and its analogue labelled at leucyl-carbonyl carbon with ^13^C (Supporting Information, Figures S2 and S3) indicated no major change in the NMR spectra with temperature ruling out any important shift of the equilibrium ratio of 2′- and 3′-isomers or even the occurrence of a stabilized tetrahedral hemiorthoester structure unable of undergoing hydrolysis (Förster et al. 1994). In the same way, the state of ionization of the starting ester is not likely to change significantly with temperature since at pH 6.5 the amino group is already mainly in a protonated state owing the p*K*_a_ value of 7.2 determined kinetically (Supporting Information) and consistent with the literature value of 7.5 at 37 °C for valyl-tRNA (Schuber and Pinck 1974). Other reasons could be responsible for the effect of temperature since interactions with the neighboring phosphate anion and protonated amine may induce differences in the stabilization of the ground state (neutral ester) and the transition state (reached from an anionic tetrahedral intermediate).

Anyway, the role of temperature in the efficiency of this reaction must be emphasized. It is worth noting that eutectic phases in ice matrices have been shown as media in which nucleotide monomers can be concentrated, thus facilitating polymerization (Monnard et al. 2003). A similar facilitation combined with the unusual kinetic stability of the aminoacyl ester could make the formation of covalent assemblies containing the structural linkage of co-polymer **1** much easier at low temperatures. The unusual influence of temperature is a consequence of the kinetic stability of RNA aminoacyl esters that was identified here to increase substantially more than that of the large majority of other reaction processes at low temperatures, which independently supports the hypothesis that the development of the common chemistry of α-amino acids and RNA, from which translation eventually emerged, should have occurred at temperatures close to 0 °C or even below and subsequently adapted to higher temperatures. It is an additional indication that low temperatures would not have been detrimental to life’s early developments and should be considered in the light of the weak young Sun’s output (Kasting 2010) and the possible occurrence of frozen environments.

### Evolutionary Significance and Perspectives

Our results on the copolymer model **3a** suggest unexpected properties of amino acid-nucleotide copolymers built on ester and phosphoramidate linkages. Firstly, in the covalent assembly, the kinetic stability of the ester bond is increased by three orders of magnitude. Secondly, we identified a plausible mechanism for the formation of aminoacylated mononucleotides from NCAs through mixed anhydrides able to undergo intramolecular transfer. Lastly, the formation of phosphoramidates was confirmed to be effective owing to the increased nucleophilicity of amines compared to alcohols in phosphoryl transfer. The chemistry disclosed here may be useful for building biologically compatible conjugates allowing to reach a wide variety of structures based on peptides and nucleic acids and having lifetimes compatible with applications in medicine. However, we will focus in this report on the relevance of copolymer structure **1** to the early development of life. Having demonstrated that oligomers based on phosphoramidate linkages between α-amino acid residues and nucleotides have strongly increased stabilities compared to esters at the 3′-end of RNAs and that their abiotic formation could not be considered as much less probable as that of “pure” RNA strands at the chemical stage of evolution suggests a role for these structures in the emergence of life that should not be underestimated. This statement is worth to be analyzed in the light of recent studies pointing toward a role for phosphoramidate intermediates in the formation of both peptide (Jauker et al. 2015; Griesser et al. 2017a, b) and internucleotidic linkages (Ni et al. 2009) or that of phosphoramidate-based activating agents (Gibard et al. 2017). It should also be related to the observation that a single chemical network can lead to the formation of both amino acid and nucleotides building blocks from a photocatalytic redox cycle (Patel et al. 2015; Sutherland 2017). The presence of both kinds of monomers at the same location is consistent with the formation of the copolymers considered here. It also accounts for the selection of adenylates and aminoacyl esters of nucleotides before the emergence of translation, which would otherwise be puzzling. Considering this fact in the light of the later developments of translation suggests that the contribution of copolymer structures to the process should be considered more thoroughly since it may point toward the nature of the evolutionary driving force that led to the development of ribosomal synthesis. The advantage of this approach lies in the chemical consistency of the presence of nucleotide and α-amino acid copolymers with the later evolution of replication of nucleic acids and translation.

**Scheme 4:**
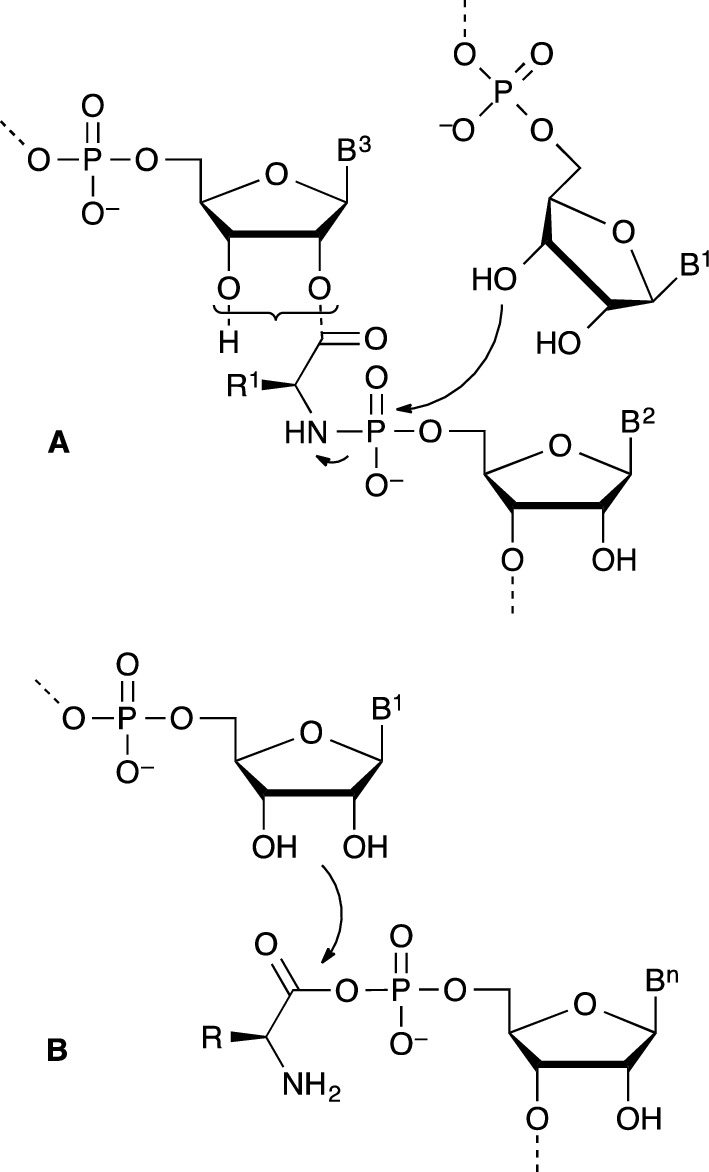
Hypothetic mechanisms through which amino acid – nucleotide phosphoramidates could serve as phosphoryl transfer agents (**a**) and of aminoacyl moieties could be transferred from mixed anhydrides (**b**) thanks to a precise positioning of reacting groups

Though the behavior of these copolymers will need deeper investigation, we can conceive that the folding behavior of α-amino acid residues as well as the introduction of functional diversity from their side-chains is likely to open extended possibilities of molecular recognition. They may additionally have played a dynamic role at least as intermediates with lifetimes reaching several weeks at 20 °C and more at lower temperature values that could match the generation time of early entities. Many possibilities can be considered including different combinations of co-oligomer sequences within larger macromolecules based on oligonucleotides or peptides that cannot be limited at this stage. At the difference of phosphodiester or peptide linkages, the copolymer structure should present a residual reactivity suggesting a transient role, several hypotheses can be considered with respect to that dynamic participation to a nucleotide metabolism owing to the electrophilic character of both the ester and the phosphoramidate linkages. The first one resulting from the reaction of the ester moiety obviously predates ribosomal synthesis through the formation of non-coded peptides initiated by the free amino group of the aminoacylated 3′-end. The second one, eventually leading to phosphodiesters (Scheme 4), is deduced from the observation that phosphoramidates correspond to reactive species in phosphoryl transfer (Ni et al. 2009) undergoing hydrolysis but that have also been identified as potential precursors of pyrophosphates by reaction with other phosphoryl groups and of phosphodiesters by reaction with alcohols. Interestingly certain amino acid phosphoramidates have also been used as substrates for polymerases (Adelfinskaya et al. 2007).

An additional hypothesis, not incompatible with the preceding ones can be put forward. The aminoacylation process occurring through intramolecular transfer in aminoacyl adenylates, which is stereoselective and highly efficient at low pH (below pH 6) provided that carbon dioxide remains at low concentrations (Wickramasinghe et al. 1991), can produce limited overall yields only. We additionally demonstrated that NCAs can serve as aminoacylating agents working at low concentrations where their polymerization could be controlled provided that the target nucleotide bears a phosphoryl group present as a di-anion. The efficient intramolecular transfer observed from the 5′-AMP adduct with NCAs as well as the earlier observation of similar processes from 3′-phosphorylated nucleotides (Biron et al. 2005) opens the possibility of more sophisticated pathways with improved yields (Scheme 4). Certain strands of amino acid-ribonucleotide copolymers in which the carbonyl group of the mixed anhydride formed at the 5′-phosphorylated end is located in close proximity to a 3′-end could greatly facilitate the intramolecular transfer owing to a favorable folded structure or supramolecular assembly constituted by complementary strands as already observed from RNA mini-helices (Tamura and Schimmel 2004). Combined with the earlier observation of an aminoacylation process of 3′-phosphorylated nucleosides by NCAs involving the intramolecular transfer from a mixed anhydride intermediate, this observation suggests a wider role of NCAs in aminoacylation through mixed anhydrides intermediates from pending phosphate groups of RNA. The role of adenylates in biological aminoacylation could therefore have originated in the activity of an oligonucleotide or a copolymer having a phosphate pending group, for instance at the 5′-end, serving as a handle capable of transferring aminoacyl groups formed by reaction with NCA to the 3′-end of the folded structure or of that of another strand bound by Watson–Crick base-pairing. From an evolutionary perspective, it could be considered that the advent of protein aminoacyl-tRNA synthetases has removed any catalytic utility to the RNA chain and that the AMP moiety of adenylate mixed anhydrides is just the remnant of that early ribozyme from which all the bases that had been made useless by the protein catalytic activity were discarded by natural selection. It is worth noting that at least some free 5′-phosphoryl groups are present in the sequence of the short aminoacylation ribozymes identified by the group of Yarus (Turk et al. 2010, 2011), which suggests that short oligonucleotides could play a role in aminoacylation by NCA as well. As short oligonucleotides, copolymers having increased folding abilities could also act as catalysts for the aminoacylation of the copolymers devised here. This latter possibility opens a new systems chemistry perspective owing to the acid lability of phosphoramidates that may then turn into an advantage because the cleavage of one strand into two shorter ones would lead to a twofold increase in the available aminoacylated 3′-ends and therefore to a possibility of autocatalytic growth of these short oligomers.

## Conclusions

The results described here support the view that the biochemistry of translation evolved from earlier stages in which the reproduction of chemical entities could be based on the interaction of α-amino acids and nucleotides rather than from a stage in which life was based on one of these families of copolymers only (Borsenberger et al. 2004). A large variety of mixed structures based on ribonucleotide and amino acid monomers can be considered as plausible in an early life context. The fact that they are not observed in biochemistry should not be used to discard them without considering their potential in early life from which peptidyl-tRNA may still represent a universal remnant. Although the historical path will certainly not be reconstituted, our results obtained from an approach inspired by *systems chemistry* (Kindermann et al. 2005; Ludlow and Otto 2008) give an indication of how rudimentary chemical processes can be associated with this aim. They additionally suggest that low temperatures were favorable to the development of translation illustrating how chemical kinetics can therefore constitute an original marker that constrained molecular evolution and thus the more probable environmental conditions in which the biochemical pathway emerged. At least, these results can legitimate a future search for networks of reactions derived from the chemistry presented here and that could be capable of potentially overcoming the improbability of complex structure formation owing to an ability to self-reproduce and therefore to grow exponentially and become persistent (Pross 2016; Pascal et al. 2013; Pascal and Pross 2015).

## Electronic supplementary material

Below is the link to the electronic supplementary material.

Supplementary material 1 (PDF 1340 KB)
