# Supplementary material for: The Chemical Likelihood of Ribonucleotide-α-Amino acid Copolymers as Players for Early Stages of Evolution
Source: J Mol Evol. 2019 Feb 20;87(2):83–92. doi: 10.1007/s00239-019-9887-7 (PMC6443614; doi:10.1007/s00239-019-9887-7)

The chemical likelihood of ribonucleotide – α-amino acid copolymers as players for early stages of evolution

Ziwei Liu · Ghinwa Ajram · Jean-Christophe Rossi · Robert Pascal

Supporting Information

General procedures

Amino acids and 1-ethyl-3-(3-dimethylaminopropyl)carbodiimide (EDC) were purchased from Bachem, deuterated solvents were purchased from Euriso-Top, 1,1'-carbonyldiimidazole (CDI) was purchased from Acros. The rest of the reagents and solvents were purchased from Sigma-Aldrich. All compounds were used without further purification unless otherwise mentioned. Tyr(Me)-NCA was prepared as published earlier (Liu et al. 2014). In all the experiments, the pH was monitored using a Thermo Orion 3-STAR pH-meter with a VWR electrode. NMR spectra were recorded on Bruker Avance 300, Avance 400, Avance III 500 and Avance III 600 spectrometers. HPLC analyses were performed on a Waters Alliance 2690 system with a photodiode array detector 996 (system A) or a Thermo Scientific Dionex UltiMate 3000 Standard system including an autosampler unit, a thermostated column compartment and a photodiode array detector, using UV absorbance detection (acquisition in the 200–400 nm range) (system B). Separations were made using Thermo Scientific BDS Hypersil C18 5 µm 2.1 × 50 mm columns using gradients of solvents. Method A: system A, mobile phase: A: H_2O + 0.1% TFA, B:CH_3CN + 0.1% TFA; flow rate: 0.2 mL/min; 0 min (5% B), to 15 min (15% B), 25 min (60% B) and 26 min (100% B); Method B: system A, mobile phase: A: 5 mM triethylammonium acetate pH 6 in water, B: CH_3CN; flow rate: 0.2 mL/min; 0 min (5% B), to 15 min (15% B), 25 min (60% B) and 26 min (100% B); Method C: system B, mobile phase: A: H_2O (0.1% TFA), B: CH_3CN (0.1% TFA); flow rate 0.2 mL/min; gradient: 0 min (5% B), 10 min (10% B), 15 min (35%) , 18 min (100% B), 19 min (100% B), 21 min (100% B) ; HPLC-ESI-MS analyses were carried out on a Waters Synapt G2-S system connected to a Waters Acquity UPLC H-Class apparatus equipped with an Acquity UPLC BEH C18, 1.7mm 2.1 × 50 mm column (system C) or a Thermo Scientific BDS Hypersil C18 5 µm 2.1 × 50 mm column (system D); separations were made using Thermo Scientific BDS Hypersil C18 5 µm 2.1 × 50 mm columns using gradients of solvent. Method D: system C, mobile phase: A: H_2O + 0.01% formic acid, B: CH_3CN + 0.01% formic acid; flow rate: 0.5 mL/min; linear gradient 0% to 100% B over 3 min. Method E: system D, mobile phase solvent A: H_2O (0.1% formic acid), B: CH_3CN (0.1% formic acid); flow rate 0.2 mL/min; gradient:

0 min (5% B), 10 min (10% B), 15 min (35%) , 18 min (100% B), 19 min (100% B), 21 min (100% B).

Adenosine 5'-methylphosphate (Me-5'-AMP). The methylation of 5'-AMP was carried out according to a published procedure (Smith et al. 1958) by reaction of adenosine 5'-phosphate with dicyclohexylcarbodiimide in methanol. 5'-AMP hydrate (0.95 mmol, 347 mg) was dissolved in methanol (50 mL) containing *N,N*-diisopropylethylamine (DIEA 2 mmol 348 µL) and dicyclohexylcarbodiimide (DCC 5 mmol, 1.032 g). After three days at room temperature the solvent was evaporated and the residue dissolved in water containing sodium hydroxide (2.2 mmol). The solution was filtered, washed by ether, evaporated to dryness. The residue was dissolved in the minimum of methanol and precipitated with acetone. The white precipitate (sodium salt) was washed by acetone and dried in vacuum (yield 386 mg). ^{1}H NMR (300 MHz, D_2O, 20 °C) δ 8.36 (s, 1H), 8.17 (s, 1H), 6.02 (d, J = 5.6 Hz, 1H), 4.44 – 4.37 (m, 1H), 4.28 (s, 1H), 4.07 – 3.97 (m, 2H), 3.43 (d, J = 10.8 Hz, 3H). ^{31}P NMR (121 MHz, D_2O) δ 1.64 (s). HRMS (ESI negative mode): calcd for $C_{11}H_{15}N_5O_7P^-$, 360.0709; found 360.0708.

General procedure for preparing amino acylated mononucleotides 4a–f (mixture of 2' and 3' regioisomers) (Liu et al. 2016; Weber and Orgel 1978). The *N-tert*-butyloxycarbonyl (Boc) amino acid (0.5 mmol) was dissolved in dimethylformamide (DMF, 0.125 mL). 1,1'-carbonyldiimidazole (CDI, 89.2 mg, 0.55 mmol) was added to the solution and allowed to react for 5 min at room temperature. The resulting solution of the amino acid imidazolide was added to a solution of nucleotide (0.26 mmol) in water (0.25 mL). After reacting for one night, the solution was adjusted pH to 2 by HCl, and then washed by ethyl acetate (0.5 ml) three times (the extraction of unreacted Boc-amino acid by ethyl acetate was avoided for the preparation of **4a**). The mononucleotide aminoacyl ester was isolated from the aqueous phase by preparative HPLC C18 column with the gradient: mobile phase: A: H_2O + 5 mM triethylamine and acetic acid at pH 6.5, B: acetonitrile; flow rate: 9 mL/min; 0 min (10% B), to 21 min (60% B), and 22 min (100% B). The solvent was removed by freeze-drying. The Boc-protecting group was removed by trifluoroacetic acid (TFA, 1 mL) for 5 min. The precipitate was collected by centrifugation after adding diethyl ether, washed by dichloromethane twice and diethyl ether once and then dried under

vacuum. The products (white powders, obtained in usually 20% yield) were stored at –20 °C.

2’(3’)-*O*-Methyl-tyrosyl adenosine 5’-methylphosphate, **4a**: ^{1}H NMR (300 MHz, D_2O, 20 °C) 3’-regioisomer: δ 8.58 (s, 1H), 8.46 (s, 1H), 7.33 (d, *J* = 8.5 Hz, 2H), 7.05 (d, *J* = 8.5 Hz, 2H), 5.89 (d, *J* = 7.2 Hz, 1H), 5.59 – 5.49 (m, 1H), 5.01 (dd, *J* = 7.1, 5.4 Hz, 1H), 3.79 (s, 3H), 3.59 (d, *J* = 10.8 Hz, 3H), other signals for this compound were mainly obscured; 2’-regioisomer: δ 8.57 (s, 1H), 8.42 (s, 1H), 7.20 (d, *J* = 8.5 Hz, 2H), 6.86 (d, *J* = 8.5 Hz, 2H), 6.31 (d, *J* = 4.4 Hz, 1H), 5.79 (t, *J* = 4.9 Hz, 1H), 4.37 (d, *J* = 2.5 Hz, 1H), 3.79 (s, 3H), 3.58 (d, *J* = 10.8 Hz, 3H), other signals for this compound were mainly obscured.

2’(3’)-Leucyl adenosine 5’-methylphosphate, **4c**: ^{1}H NMR (300 MHz, D_2O, 20 °C) 3’-regioisomer: δ 8.59 (s, 1H), 8.42 (s, 1H), 6.20 (d, *J* = 6.6 Hz, 1H), 5.64 (dd, *J* = 4.8, 2.2 Hz, 1H), 5.06 (t, *J* = 6.0 Hz, 1H), 4.59 (s, 1H), 3.56 (d, *J* = 10.8 Hz, 3H), 2.11 – 1.64 (m, 3H), 0.96 (dt, *J* = 6.4 Hz, 6H), other signals for this compound were mainly obscured, 2’-regioisomer: δ 8.56 (s, 1H), 8.41 (s, 1H), 6.40 (d, *J* = 3.8 Hz, 1H), 5.75 (t, *J* = 4.6 Hz, 1H), 4.87 – 4.83 (m, 2H), 3.56 (d, *J* = 10.8 Hz, 3H), 1.93 (m, 3H), 0.94 (t, *J* = 5.0 Hz, 6H), other signals for this compound were mainly obscured. ^{1}H NMR (600 MHz, D_2O at 2 °C and 25 °C) Figure S2.

^{13}C-Labeled 2’(3’)-leucyl adenosine 5’-methylphosphate, **[1-^{13}C]-4c**: The compound was prepared from Boc-Leu-OH-1-^{13}C monohydrate**.** ^{13}C NMR (151 MHz, D_2O, 2 °C) Figure S3. The absence of a peak at ca. 60–65 ppm (Förster et al. 1994) in the ^{13}C-NMR spectrum at 2 °C (as well as at 25 °C, not shown) demonstrates that the possible occurrence of a tetrahedral hemi-orthoester isomer involving a bond with both 2’- and 3’-hydroxyl groups is not susceptible of having a significant influence on the kinetics of hydrolysis.

2’(3’)-Leucyl adenosine 5’-phosphate, **4d**: ^{1}H NMR (300 MHz, D_2O, 20 °C) 3’-regioisomer: δ 8.53 (s, 1H), 8.32 (s, 1H), 6.13 (d, *J* = 6.9 Hz, 1H), 5.58 (dd, *J* = 5.3, 2.1 Hz, 1H), 5.03 – 4.94 (m, 1H), 4.53 (s, 1H), 4.08 (dd, *J* = 4.7, 2.8 Hz, 2H), 1.83 – 1.58 (m, 3H), 0.92 (dd, *J* = 12.2, 5.5 Hz, 6H), other signals for this compound were mainly obscured; 3’-regioisomer: ^{1}H NMR (300 MHz, D_2O, 20 °C) δ 8.50 (s, 1H), 8.29 (s, 1H), 6.32 (d, *J* = 4.0 Hz, 1H), 5.72 – 5.65 (m, 1H), 4.76 (dd, *J* = 9.3, 3.7 Hz, 1H),

4.33 (d, *J* = 2.6 Hz, 1H), 4.08 (dd, *J* = 4.7, 2.8 Hz, 2H), 1.99 – 1.83 (m, 3H), 0.90 – 0.84 (m, 6H) other signals for this compound were mainly obscured.

(*N*-2'(3')-*O*-Methyl-tyrosyl adenosine 5'-methylphosphate)-5'-adenosine phosphoramidate, 3a. The 2'(3')-*O*-methyl-L-tyrosine ester **4a** (0.07 mmol), 5'-AMP (49 mg, 0.14 mmol) and EDC (537 mg, 2.8 mmol) were dissolved in 2-(N-morpholino)ethanesulfonic acid (MES) buffer (7 mL, 100 mM of MES, pH 6.5). The reaction mixture was maintained at 4°C for 10 days. Then the product was injected directly on a preparative HPLC column and eluted with the gradient as follows. Mobile phase: A: H_2O + 5 mM triethylamine and acetic acid at pH 5.5, B: acetonitrile; flow rate: 9 mL/min; 0 min (0% B), to 5 min (0% B), to 20 min (23% B) and 21 min (100% B). The fractions containing the product were contaminated by *N*-ethyl-N'-(dimethyl)aminopropyl urea present as a counter-ion, they were treated on column of Dowex 50 ion exchange resin (10 mL) previously equilibrated with 1 M triethylammonium acetate (pH 6). The column was eluted with 1 mM triethylammonium acetate (100 mL). The resulting solution was freeze-dried to yield the product as a white powder (40 mg, theoretically 74.9 mg for **3a** bis-triethylammonium salt) that was then conserved at –18°C. ^{1}H NMR (400 MHz, D_2O, 20°C) Figure S4. COSY (400 MHz, D_2O, 20°C, Figure S5); ^{13}C NMR (100.6 MHz, D_2O, 20°C, Figure S6); ^{31}P NMR (162 MHz, D_2O, 20 °C, Figure S7) δ 6.13 (s), 5.81 (s), 1.30 (s), 1.44 (s); HRMS (ESI+): *m/z* calcd for $C_{31}H_{40}N_{11}O_{15}P_2^+$ $[M + H]^+$ 868.2181; found, 868.2184.

***O*-Methyl-L-tyrosine methyl ester adenosine-5'-phosphoramidate, 5e**. 5'-AMP (340 mg, 0.93 mmol) and the TFA salt of H-Tyr(Me)-OMe (Beaufils et al. 2016) (100 mg, 0.48 mmol) were dissolved in 100 mM imidazole buffer (pH 6.5, 5 mL), and 1N NaOH (1.3 mL) was added for adjusting the pH to 7. Then EDC (375.8 mg, 1.96 mmol) was added and the mixture was stirred for five days at room temperature. The mixture was freeze-dried and then dissolved in water (1 mL). The product was separated and purified by preparative HPLC using following gradient: mobile phase: A: H_2O + 5 mM triethylamine and acetic acid at pH 6.5, B: acetonitrile; flow rate: 9 mL/min; 0 min (0% B), to 30 min (30% B), and 33 min (100% B). ^{1}H NMR (300 MHz, D_2O) δ 8.26 (s, 1H), 8.12 (s, 1H), 6.78 (d, *J* = 8.6 Hz, 2H), 6.50 (d, *J* = 8.7 Hz, 2H), 5.95 (d, *J* = 5.7 Hz, 1H), 4.64 (t, *J* = 5.5 Hz, 1H), 4.36 – 4.28 (m, 1H), 4.21 – 4.14 (m,

1H), 3.77 (dd, *J* = 10.2, 7.4 Hz, 2H), 3.68 (q, *J* = 7.0 Hz, 1H), 3.57 (s, 3H), 3.52 (s, 3H), 2.64 (dt, *J* = 9.8, 4.8 Hz, 2H).

***O*-Methyl-L-tyrosine adenosine-5'-phosphoramidate, 5a**. The methyl ester **5e** was hydrolyzed in methanol/water (1:2 v/v) solution after addition of 1N NaOH (1.5 eq.) for 4 hours, and then the solvent was evaporated. Then the pH was adjusted to pH 7 by adding HCl. Then the solution was freeze-dried and this solid was used without further purification for kinetic studies. ^{1}H NMR (300 MHz, D_2O) δ 8.31 (s, 1H), 8.08 (s, 1H), 6.86 (d, *J* = 7.6 Hz, 2H), 6.55 (d, *J* = 8.4 Hz, 2H), 5.82 (s, 1H), 4.66 – 4.60 (m, 2H), 4.18 (s, 1H), 4.13 (s, 1H), 3.77 (s, 2H), 3.66 – 3.61 (m, 1H), 3.60 (s, 3H), 2.63 (dd, *J* = 13.9, 7.7 Hz, 2H). Other signals for this compound were mainly obscured. ^{31}P NMR (121 MHz, D_2O) δ 6.95 (s).

L-Leu-NCA. The *N*-carboxyanhydride was prepared by reaction of H-L-Leu-OH with triphosgene (Daly and Poche 1988) (yield 24%). ^{1}H NMR (400 MHz, $CDCl_3$) δ 6.69 (s, 1H), 4.28 (dd, J = 9.1, 3.4 Hz, 1H), 1.85 – 1.56 (m, 3H), 1.01 – 0.85 (m, 6H). ^{13}C NMR (101 MHz, $CDCl_3$) δ 169.87 (s), 152.84 (s), 56.18 (s), 40.84 (s), 25.08 (s), 22.70 (s), 21.53 (s).

L-Leucyl-adenylate, 6d. The mixed anhydride was prepared using a method earlier used for the preparation of L-valyl-adenylate (Biron et al. 2005). An aq. soln. of the free acid of 5'-AMP (365 mg, 1 mmol) was converted into a mixture of mono- and di-anionic forms by adding DIEA (190 µL, 1.1 equiv). The soln. was frozen and lyophilized. Independently, *N*-Boc-L-Leu-OH (426.5 mg, 1.84 mmol) was converted into symmetrical anhydride by reaction with DCC (190 mg, 0.92 mmol) for 2 h in CH_2Cl_2 (20 mL) at r.t. The *N,N'*-dicyclohexylurea by-product was removed by filtration and the soln. was concentrated in vacuum without heating, the residue was diluted with DMF and added to the 5'-AMP-DIEA salt (0.207g, 0.42 mmol) with further DIEA (73 µL, 0.42 mmol). The reaction mixture was stirred for 2 h at r.t. and the product was precipitated by addition of Et_2O. The precipitate was collected by centrifugation and the supernatant was removed. The residue was washed with Et_2O and dried in vacuum. The resultant white residue was dissolved in TFA (1 mL) and allowed to react at r.t. for 30 min. Excess Et_2O was added and the precipitate was collected by centrifugation. The solid was further washed with Et_2O, twice by CH_2Cl_2 and again with Et_2O before being dried in vacuum. The product containing residual 5'-AMP was

used without further purification. ^{1}H NMR (300 MHz, D_2O) δ 8.60 – 8.43 (m, 1H), 8.35 (t, J = 7.6 Hz, 1H), 6.13 ((dd, J = 10.7, 6.9 Hz, 1H, H-C(1')), 4.44 (t, J = 4.5 Hz, 1H), 4.30 (t, J = 9.9 Hz, 1H), 4.26 – 4.13 (m, 1H, $(CH_3)_2CH$-CH_2-C*H*-CO-), 4.13 – 3.99 (m, 2H), 1.85 – 1.53 (m, 3H, $(CH_3)_2$*CH*-*CH_2*-CH-), 0.85 – 0.68 (m, 6H, (*CH_3*)$_2$CH). ^{31}P NMR (121 MHz, D_2O) δ –8.28. Data for residual AMP: ^{1}H NMR (300 MHz, D_2O) δ 8.55 (s, 1H), 8.35 (s, 1H), 6.12 (d, J = 5.4 Hz, 1H, H-C(1')),), 4.68 (s, 1H), 4.44 (dd, J = 5.0, 4.0 Hz, 1H), 4.35 – 4.30 (m, 1H), 4.13 – 4.01 (m, 2H, H-C(5'). ^{31}P NMR (121 MHz, D_2O) δ 0.17

Kinetics of hydrolysis of the co-trimer model 3a. The kinetics of hydrolysis of the co-trimer **3a** (1 mM concentration) were monitored in solutions maintained in a room thermostated at 25°C in the presence of buffers prepared according to table S1. For slow reactions at pH values between 5.5 and 7.5, one drop of toluene was added to avoid the development of bacteria just before starting experiments. The reaction progress was monitored by withdrawing 15 µL samples that were diluted to 1.5 mL before HPLC analysis (method A). Values of pseudo-first-order rate constants k_{obs} were calculated by curve-fitting to an exponential decrease using a least-squares method. The calculated curves of Figure 3 (main text) were determined by curve fitting using a least-squares method considering a rate law involving terms corresponding to pH-independent ($k_0 = 6 \times 10^{-8}$ s^{-1}), specific acid catalysis ($k_H = 5.5 \times 10^{-3}$ M^{-1}s^{-1}) and specific base catalysis ($k_{OH} = 1.4$ M^{-1}s^{-1}).

$$k_{obs} = k_0 + k_H [H^+] + k_{OH} [OH^-]$$

The values of $[H^+]$ and $[OH^-]$ used for the calculation were determined from the value of pH and the ionic product of water ($K_w = 10^{-14}$).

Table S1. Buffer solutions used for different pH values.

pH value	Buffer
2	10 mM HCl + 90 mM NaCl
3	50 mM HCOOH adjusted by NaOH
4	50 mM CH_3COOH adjusted by NaOH
5.5 – 6.5	50 mM MES [a] adjusted by NaOH
7.5	50 mM MOPS [b] adjusted by NaOH
8.5 – 9.5	50 mM PIPPS [c] adjusted by NaOH
8.5–10	25 mM sodium tetraborate adjusted by addition of HCl or NaOH

[a] MES: 2-morpholino-ethane-1-sulphonic acid; [b] MOPS: 3-morpholino-propane-1-sulphonic acid; [c] PIPPS: piperazine-*N,N'-bis*(3-propanesulfonic Acid).

Kinetics of hydrolysis of Me-5'-AMP leucyl ester 4c. The reaction of the ester (initial concentration 50 µM) were carried out in buffered solution (table S1) at usually 25°C (or alternatively at 0°C in ice baths) in thermostated flasks from which 1 mL samples were withdrawn and analysed by HPLC (Method C) or directly in vials conserved in the thermostated sample compartment of the HPLC. For the fast kinetics observed at pH values above pH 7, a sufficient volume (100 µL) of 2 M HCOOH was added to the 1 mL samples withdrawn at different time values to avoid any further evolution before analysis. Rate constants were deduced from the evolution of the total area corresponding to the peaks of 2'- and 3'-regioisomers (retention times: 10.5 min and 13.0 min) by fitting the curve to an exponential decay. No significant effect of buffer concentration (10–100 mM at a constant 100 M ionic strength) was found at pH 5.5 and 6.5. The rate constants displayed in figure 3 (main text) were fitted to a kinetic law involving the nucleophilic attack of HO^- on the ester **4c** bearing a neutral amine group at high pH (k^0_{OH} = 36.1 $s^{-1}M^{-1}$) and of the protonated form **4c**+H^+ (k^+_{OH} = 3.25 x 10^3 $s^{-1}M^{-1}$, pKa = 7.2) at intermediate pH values. Additionally a pH independent term was considered as significant at pH values below 5, which may represent a pH-independent hydrolysis of the protonated form (k^+_0 = 7.4 x 10^{-7} s^{-1}, or any other kinetic equivalent involving for instance adenine protonation significant below pH 4). The deviation from calculated curves observed at low pH could be related to kinetic terms associated with changes in the ionization state of the adenine and phosphate moieties.

Rate = k_{obs} ([**4c** +H^+] + [**4c**])= (k^+_0 + k^+_{OH} [OH^-]) [**4c** +H^+] + k^0_{OH} [**4c**]

Kinetics of hydrolysis of phosphoramidate 5a and its methyl ester 5e. 50 µM Solutions of phosphoramidate **5a** or its methyl ester **5e** were prepared in buffered water (15 mL, Table S1) thermostated to 25°C and the hydrolysis was monitored by performing HPLC analyses along the progress of the reaction (method C). For the faster kinetics observed below pH 4, 1 mL samples were withdrawn from the reaction medium and further evolution was blocked by addition of 0.4 M borate buffer pH 10 (200 µL). Rate constants were determined by fitting the evolution of areas of the phosphoramidate peak to an exponential decay.

Aminoacylation of nucleotides with Leu-NCA. A 1 mM buffered solution (1 mL) of nucleotide (5'-AMP or Me-5'-AMP selected as a negative control for intramolecular transfer) was continuously degased by bubbling N_2 (from which residual traces of CO_2 were removed by previously bubbling through a trap containing 1 N NaOH). Five 50 µL portions of a 100 mM solution of Leu-NCA in dry THF were added at regular intervals of time under continuous bubbling of nitrogen. Then 100 µL samples of the solution were withdrawn at different times and analysed by HPLC (method C). The chromatographic analysis demonstrated the formation of both the mixed anhydride leucyl-adenylate (retention time 3.5 min) and the 2'(3')-leucyl ester (retention times 4.1 and 6.4 min, method C) of AMP **4d** (Table S2), which was confirmed by the identity of peaks with that of the independently prepared materials. A negative control for the intramolecular transfer was carried out involving the reaction of Me-5'-AMP unable to form a mixed anhydride under similar conditions. A positive control involved the reaction of L-leucyl-adenylate **6d** (100 µM) dissolved in 1 mL of 100 mM MES buffer (pH=5.5) in an HPLC vial.

Table S2. Results of the study of the aminoacylation observed at the 2'- and 3'-hydroxyl group of nucleotide using the reaction of Leu-NCA with 5'-AMP or its methylated analogue (control) or the preformed leucyl-adenylate (Leu-5'-AMP)

Nucleotide	pH	Leu-NCA	$MgCl_2$	Addn time [a]	Analysis time [b]	Mixed anhydride	Total 2'(3')-ester
5'-AMP	5.5	5 x 5 mM	50 mM	4 h	4 h	4.41 %	0.76 %
					5 h	4.00 %	0.84 %
					20 h	0.31 %	1.92 % [c]
5'-AMP	5.5	5 x 5 mM	–	4 h	4 h	2.03 %	0.40 %
					20 h	0.14 %	0.78 %
Me-5'-AMP	5.5	5 x 5 mM	–	4 h	4 h	n.d.	n.d.
					20 h	n.d.	n.d.
5'-AMP	6.5	5 x 5 mM	50 mM	1.5 h	1.5 h	n.d. [d]	0.47 %
					2.4 h	n.d. [d]	0.57 %
5'-AMP	6.5	5 x 5 mM	–	1.5 h	1.5 h	n.d. [d]	0.59 %
					2.4 h	n.d. [d]	0.54 %
Leu-5'-AMP **6d**	5.5	n.a.[e]	–	n.a.[e]	2.43 h	43.8 %	32.3 % [f]
					3.65 h	31.4 %	35.9 % [f]

[a] Total Leu-NCA addition time; [b] reaction time (measured since the first NCA addition when applicable); [c] HPLC-MS (method E) (ESI^+): *m/z* calc for $C_{16}H_{26}N_6O_8P^+$ $[M+H^+]$ 461.1544 found 461.16 (retention time 5.2 min) and 461.15 (retention time 8.4 min); the MS spectrum also indicated the presence of small amounts of adducts involving 2 or 3 Leu residues as expected from a polymerization initiated by the esters **4d**; [d] not detected; [e] not applicable; [f] Percentage of the reacted adenylate **6d**.

References

Liu Z, Beaufils D, Rossi J-C, Pascal R (2014) Evolutionary importance of the intramolecular pathways of hydrolysis of phosphate ester mixed anhydrides with amino acids and peptides. Sci Rep 4:7440

Smith M, Moffatt JG, Khorana HG (1958) Carbodiimides. VIII. Observations on the Reactions of Carbodiimides with Acids and Some New Applications in the Synthesis of Phosphoric Acid Esters. J Am Chem Soc 80:6204–6212

Liu Z, Hanson C, Ajram G, Boiteau L, Rossi J-C Danger G, Pascal R (2016b) 5(4*H*)-Oxazolones as Effective Aminoacylation Reagents for the 3′-Terminus of RNA. Synlett 28:73–77

Weber AL, Orgel E.(1978) The formation of peptides from the 2'(3')-glycyl ester of a nucleotide. J Mol Evol 11:189-198

Förster C, Limmer S, Zeidler W, Sprinzl M (1994) Effector region of the translation elongation factor EF-Tu GTP complex stabilizes an orthoester acid intermediate structure of aminoacyl-tRNA in a ternary complex. Proc Natl Acad Sci USA 91:454–457

Beaufils D, Jepaul S, Liu Z, Boiteau L, Pascal R (2016) The Activation of Free Dipeptides Promoted by Strong Activating Agents in Water Does not Yield Diketopiperazines. Orig Life Evol Biosph 46:19–30

Daly WH, Poche D (1988) The preparation of *N*-carboxyanhydrides of amino acids using bis(trichloromethyl)carbonate. Tetrahedron Lett 29:5859–5862

Biron J-P, Parkes AL, Pascal R, Sutherland JD (2005) Expeditious, potentially primordial, aminoacylation of nucleotides. Angew Chem Int Ed 44:6731–6734

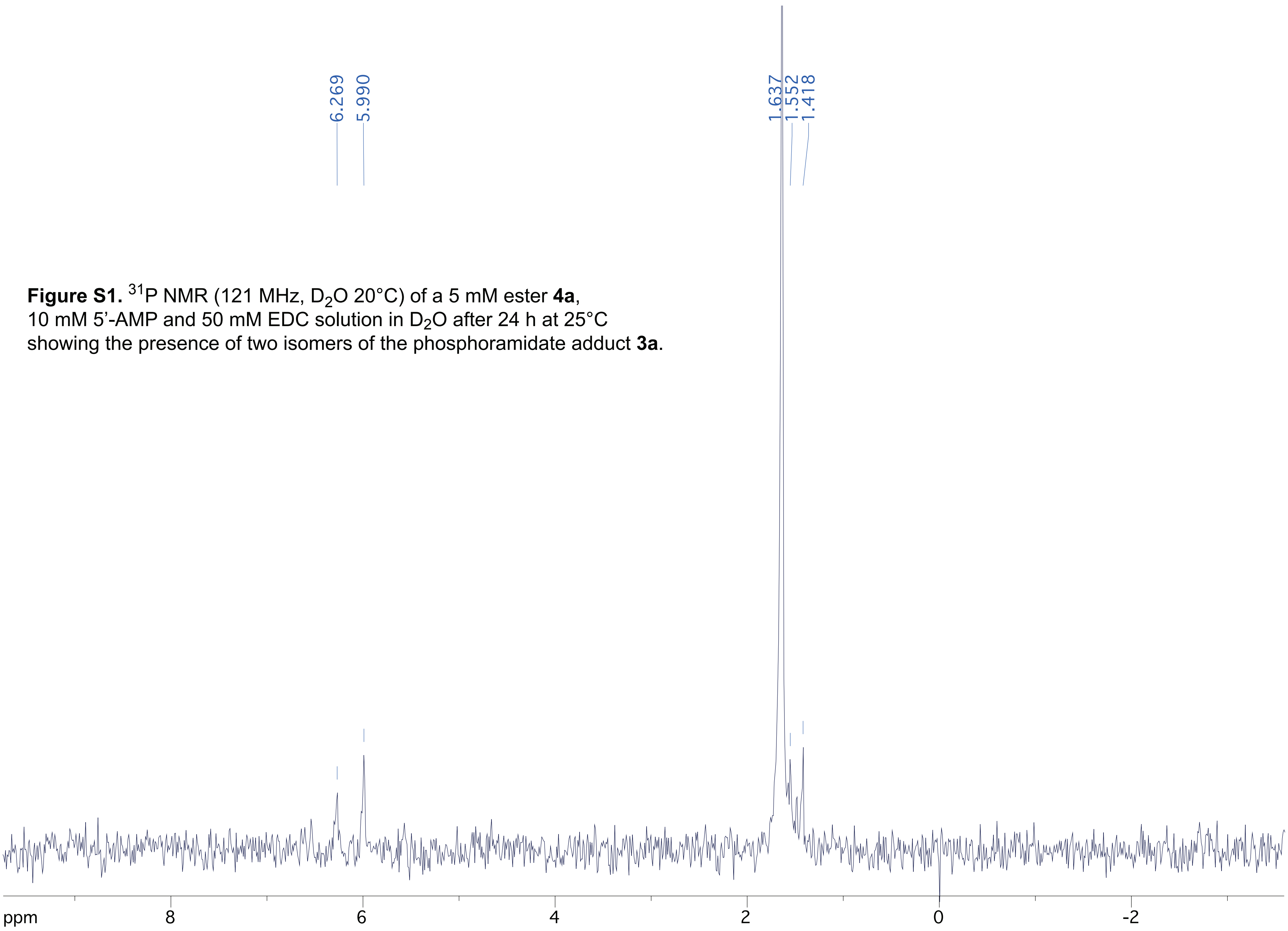

Figure S1. ^{31}P NMR (121 MHz, D_2O 20°C) of a 5 mM ester **4a**, 10 mM 5'-AMP and 50 mM EDC solution in D_2O after 24 h at 25°C showing the presence of two isomers of the phosphoramidate adduct **3a**.

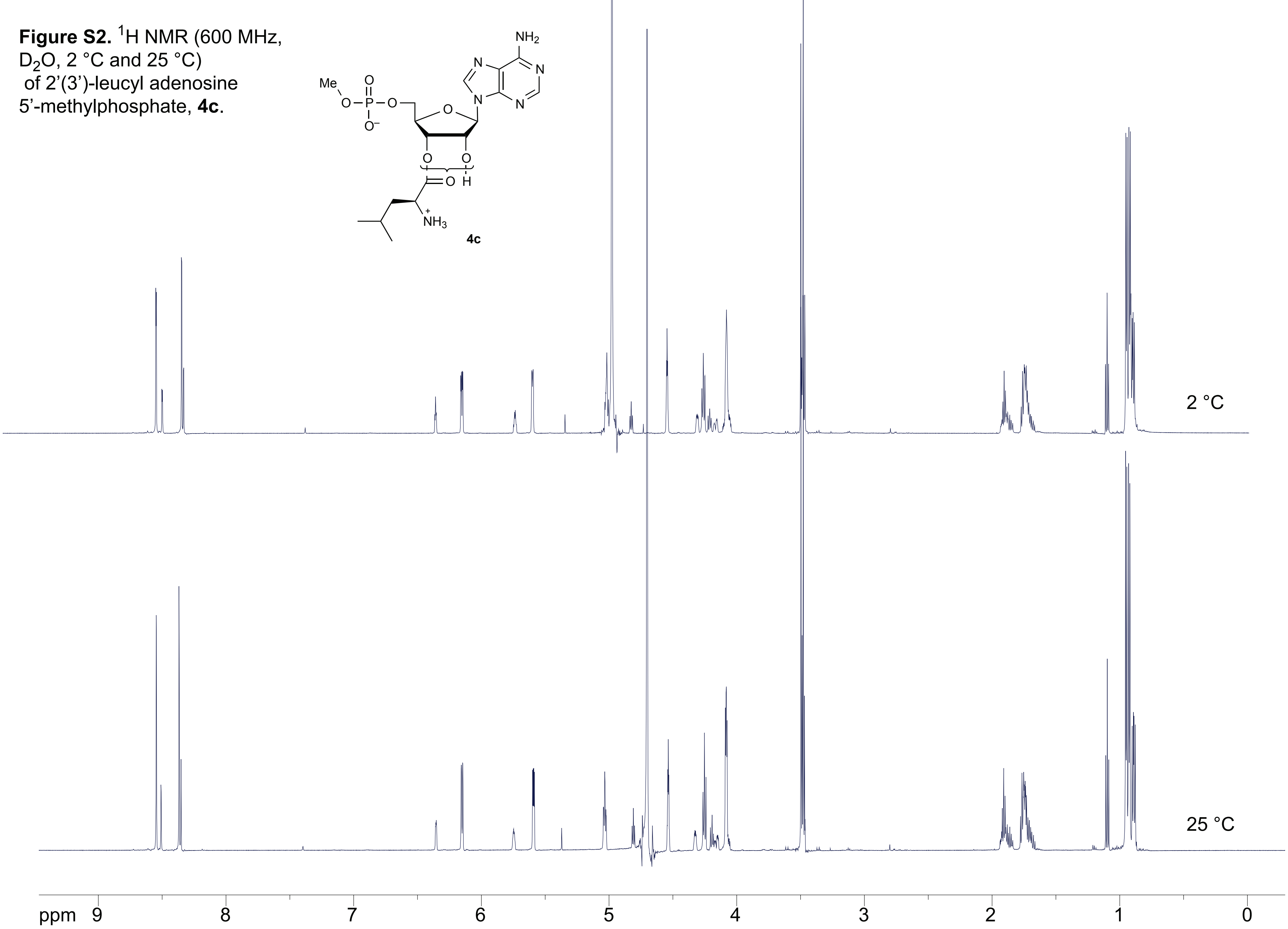

Figure S2. ^{1}H NMR (600 MHz, D_2O, 2 °C and 25 °C) of 2'(3')-leucyl adenosine 5'-methylphosphate, **4c**.

Figure S3. ^{13}C NMR (151 MHz, D_2O, 2 °C) of ^{13}C-Labeled 2'(3')-leucyl adenosine 5'-methylphosphate, **[1-^{13}C]-4c**.

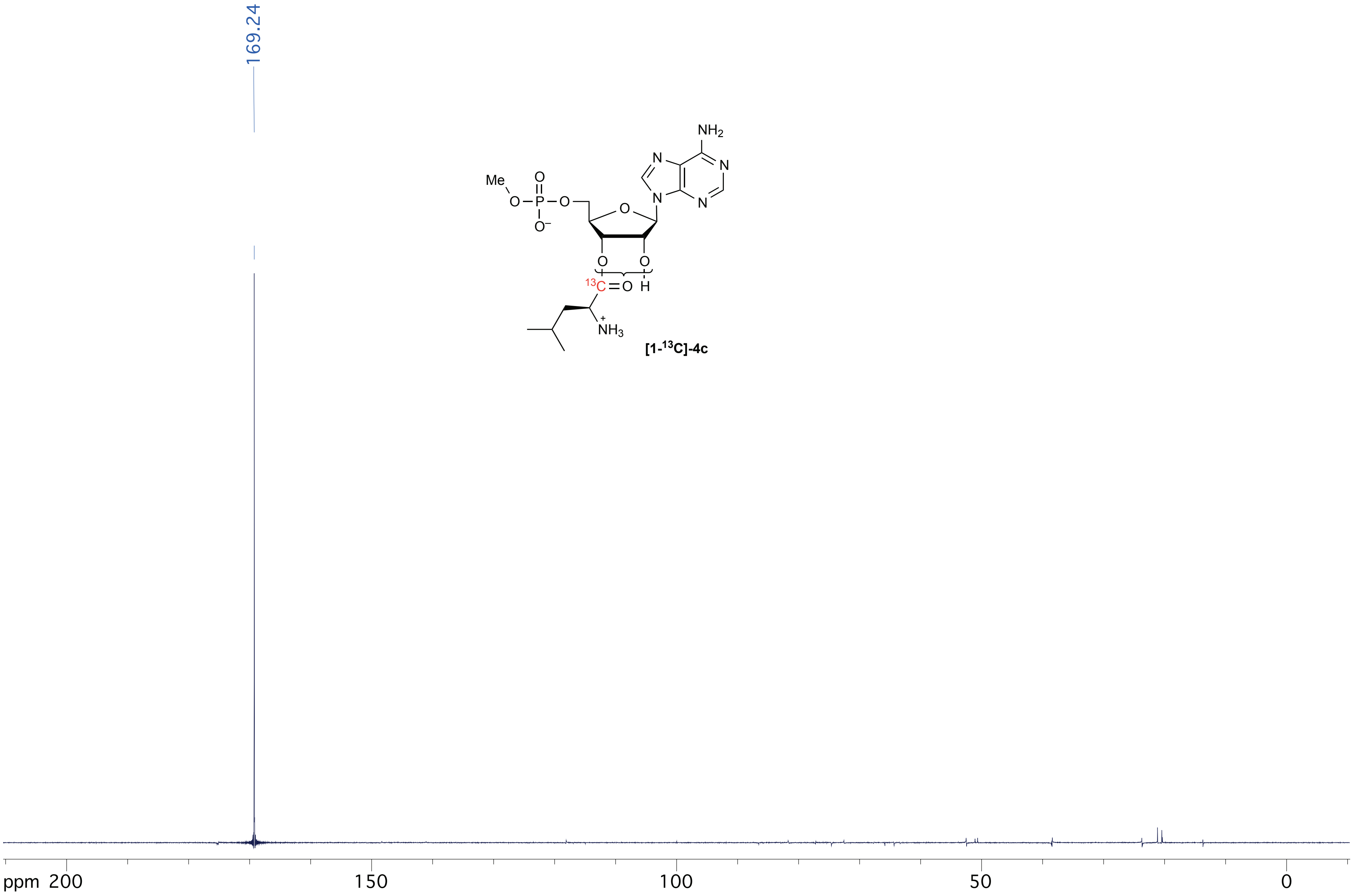

Figure S4. ^{1}H NMR (400 MHz, D_2O, 20 °C) of purified trimer model **3a** (bis-triethylamine salt).

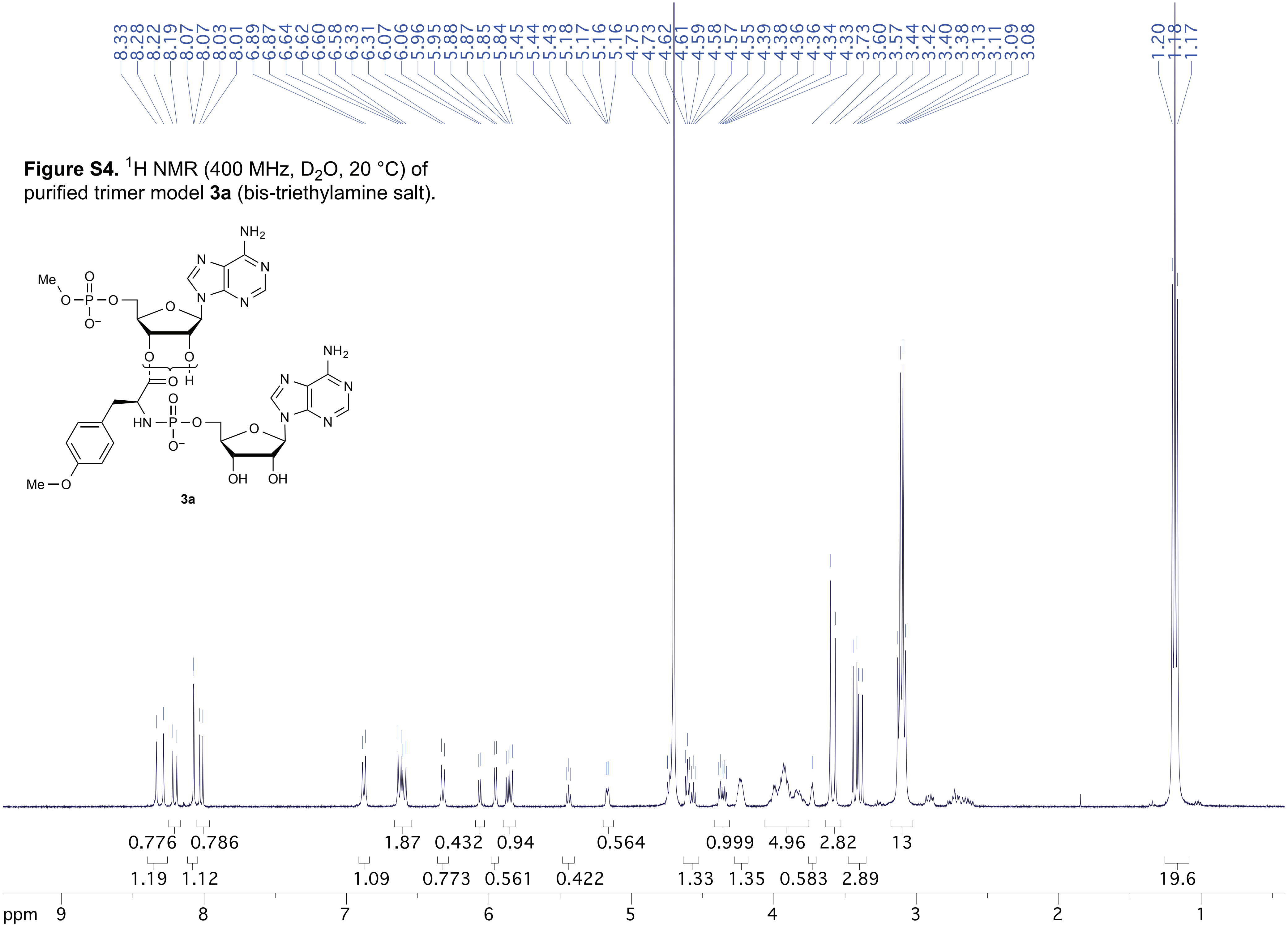

Figure S5. ^{1}H COSY NMR correlation chart (400 MHz, D_2O, 20 °C) of purified trimer model **3a** (bis-triethylamine salt).

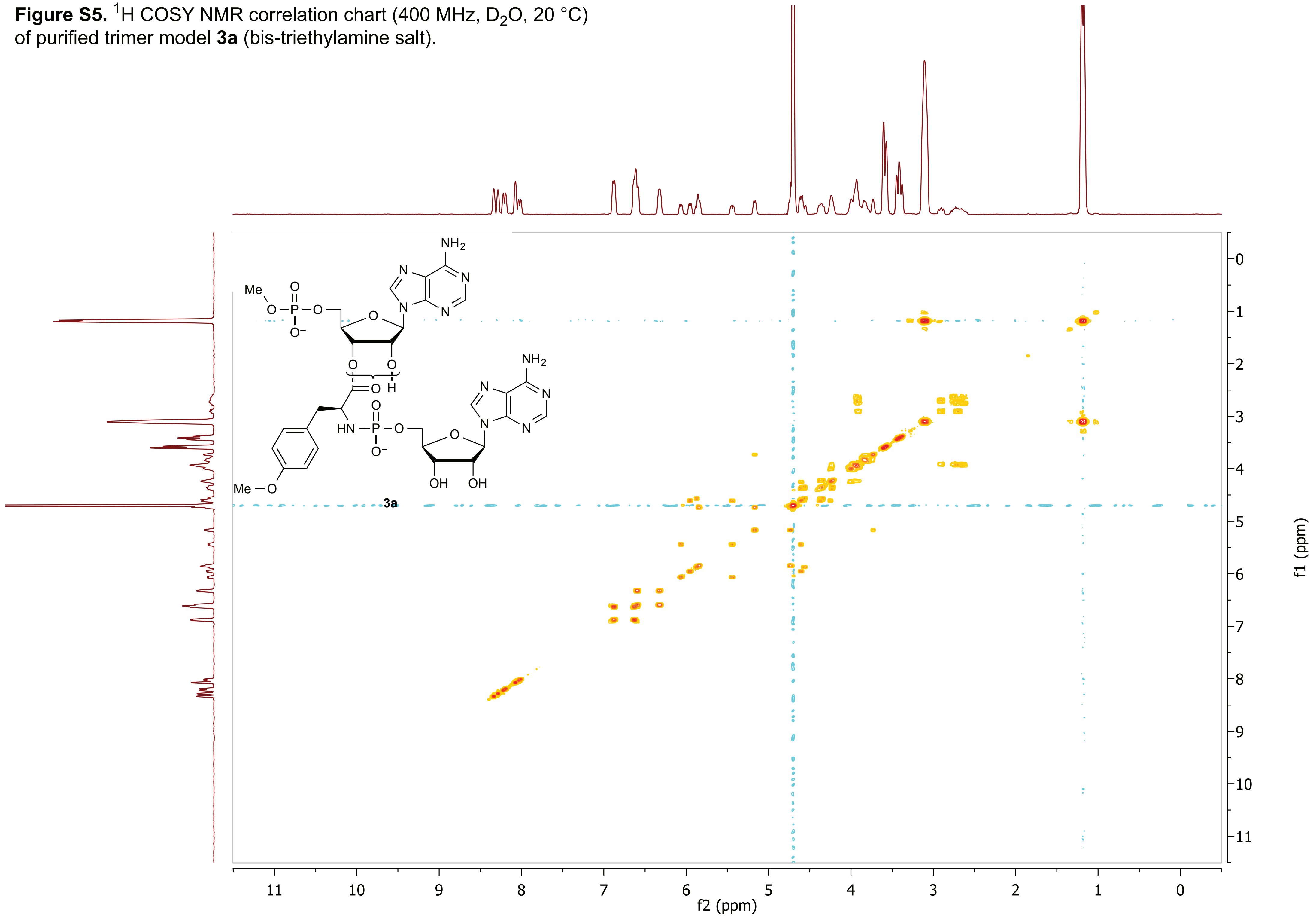

Figure S6. ^{13}C NMR (101 MHz, D_2O, 20 °C) of purified trimer model **3a** (bistriethylamine salt).

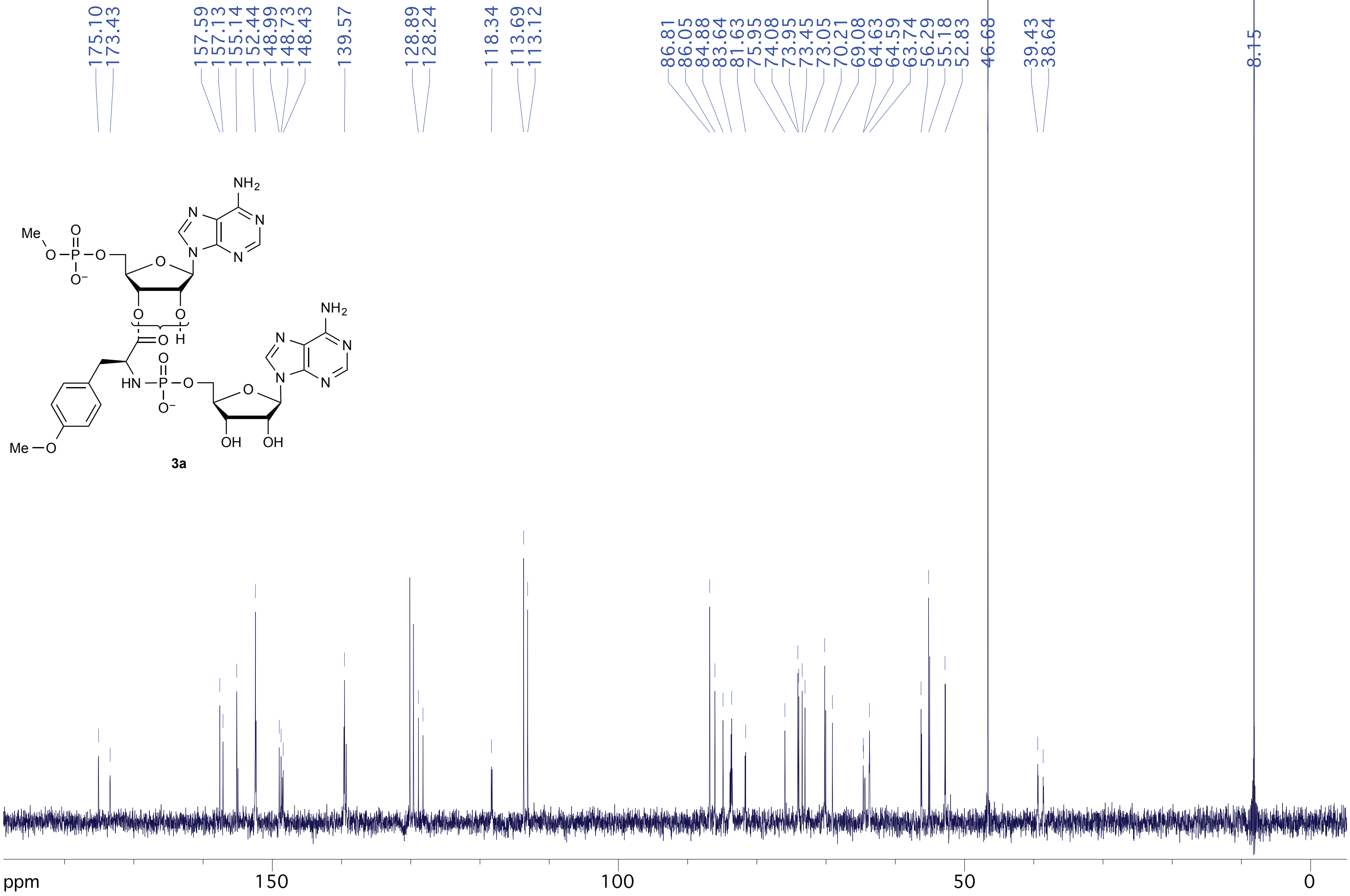

3a

Figure S7. ^{31}P NMR (162 MHz, D_2O, 20 °C) of purified trimer model **3a**.

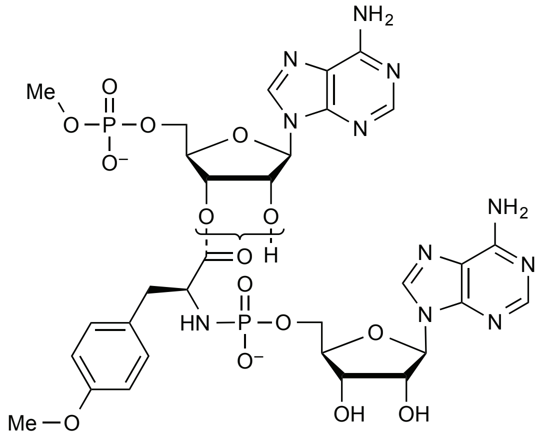

3a

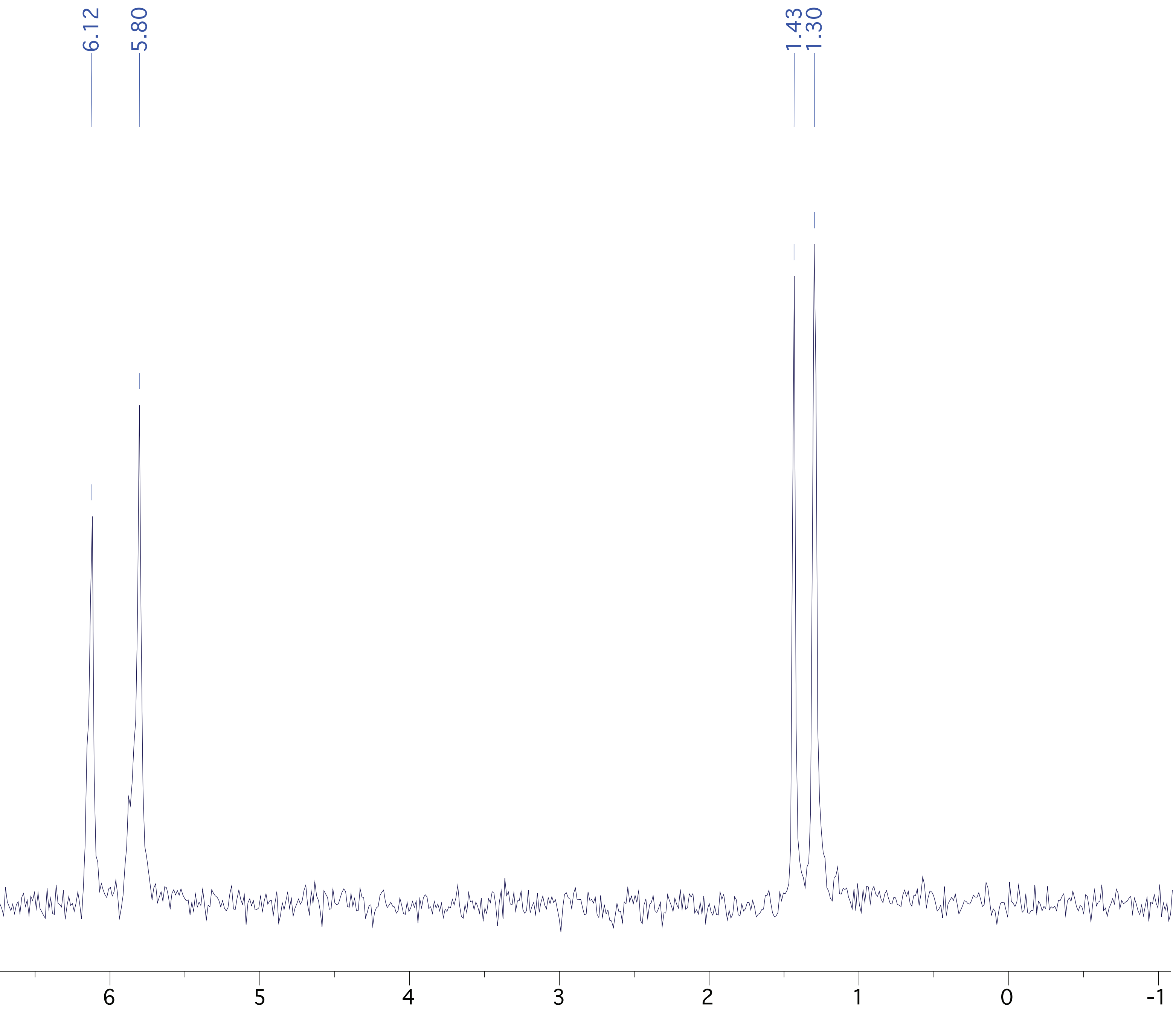

Supplement: Supplementary file 1 — Supplementary material 1 (PDF 1340 KB) [file 239_2019_9887_MOESM1_ESM.pdf]
